# Evidence of selective reporting bias in hematology journals: A systematic review

**DOI:** 10.1371/journal.pone.0178379

**Published:** 2017-06-01

**Authors:** Cole Wayant, Caleb Scheckel, Chandler Hicks, Timothy Nissen, Linda Leduc, Mousumi Som, Matt Vassar

**Affiliations:** 1 Department of Institutional Research, Oklahoma State University Center for Health Sciences, Tulsa, Oklahoma, United States of America; 2 Internal Medicine, Mayo Clinic, Scottsdale, Arizona, United States of America; 3 Internal Medicine, Oklahoma State University Medical Center, Tulsa, Oklahoma, United States of America; Hvidovre Hospital, DENMARK

## Abstract

**Introduction:**

Selective reporting bias occurs when chance or selective outcome reporting rather than the intervention contributes to group differences. The prevailing concern about selective reporting bias is the possibility of results being modified towards specific conclusions. In this study, we evaluate randomized controlled trials (RCTs) published in hematology journals, a group in which selective outcome reporting has not yet been explored.

**Methods:**

Our primary goal was to examine discrepancies between the reported primary and secondary outcomes in registered and published RCTs concerning hematological malignancies reported in hematology journals with a high impact factor. The secondary goals were to address whether outcome reporting discrepancies favored statistically significant outcomes, whether a pattern existed between the funding source and likelihood of outcome reporting bias, and whether temporal trends were present in outcome reporting bias. For trials with major outcome discrepancies, we contacted trialists to determine reasons for these discrepancies. Trials published between January 1, 2010 and December 31, 2015 in *Blood*; *British Journal of Haematology*; *American Journal of Hematology*; *Leukemia*; and *Haematologica* were included.

**Results:**

Of 499 RCTs screened, 109 RCTs were included. Our analysis revealed 118 major discrepancies and 629 total discrepancies. Among the 118 discrepancies, 30 (25.4%) primary outcomes were demoted, 47 (39.8%) primary outcomes were omitted, and 30 (25.4%) primary outcomes were added. Three (2.5%) secondary outcomes were upgraded to a primary outcome. The timing of assessment for a primary outcome changed eight (6.8%) times. Thirty-one major discrepancies were published with a *P*-value and twenty-five (80.6%) favored statistical significance. A majority of authors whom we contacted cited a pre-planned subgroup analysis as a reason for outcome changes.

**Conclusion:**

Our results suggest that outcome changes occur frequently in hematology trials. Because RCTs ultimately underpin clinical judgment and guide policy implementation, selective reporting could pose a threat to medical decision making.

## Introduction

Selective outcome reporting leading to bias in clinical trial results is a significant methodological concern in medicine [1–4]. This bias occurs when chance or selective outcome reporting rather than the intervention contributes to group differences [5]. Comparisons between outcomes reported in clinical trial registrations and those published later allow investigators to understand the extent of selective reporting bias among trialists. Thus, trial registration has played a significant role in understanding this form of bias and its effect on clinical outcomes [6].

Since 2005, the International Committee of Medical Journal Editors (ICMJE) has required that all journals within its network publish clinical trials registered in a public clinical trials registry prior to enrollment of the first patient [7–9]. Previously, only 13,153 trials were registered with ClinicalTrials.gov. This number increased to 22,714, one month after the policy took effect. Less than two years after implementation, ClinicalTrials.gov contained more than 40,000 trials [10], and to date, it contains more than 243,000 studies from 200 countries [11]. According to ICMJE, trial registration should comprise 20 items, including a description of the primary and secondary endpoints, assessment period, and funding sources, to enable stakeholders to fully investigate the validity and accuracy underlying how a trial is conducted and ultimately published [9, 12]. Despite ICMJE requirements, completeness and quality of trial registry information remain obstacles [13]. Nonetheless, information contained in trial registries allows for examining both publication and selective reporting bias [2].

The prevailing concern about selective reporting bias is the possibility of results being modified towards specific conclusions. A recent evaluation of Cochrane systematic reviews found that one-third of the included reviews contained at least 1 study for which a high suspicion of outcome reporting bias existed. Accounting for such bias changed the estimated treatment effect by 20% or more in a large number of reviews and nullified the treatment effects in others [14]. Inconsistent outcome reporting occurs when trialists add or omit outcomes, downgrade primary outcomes to secondary outcomes based on a lack of statistical significance, upgrade secondary outcomes to primary outcomes based on statistical significance, or change the outcome’s definition [15]. A systematic review of studies that evaluated the discrepancies between registered and published outcomes reported substantial variability across medical specialties in the prevalence and nature of such discrepancies, highlighting the need for further study of this issue across clinical specialties [5].

The Center for Evidence Based Medicine Outcome Monitoring Project (COMPare) currently monitors outcome reporting discrepancies for all trials published in *New England Journal of Medicine*, *Journal of the American Medical Association*, *The Lancet*, *Annals of Internal Medicine*, and *British Medical Journal* [16], notifies the journal that published the trial, and makes these discrepancies publically available. In this study, we evaluate randomized controlled trials (RCTs) published in hematology journals, a group in which selective outcome reporting has not yet been explored. Additionally, we include information from correspondence with authors of trials with major discrepancies detailing reasons that explain the changes in outcomes.

## Materials and methods

Our primary goal was to examine discrepancies between the reported primary and secondary outcomes in registered and published RCTs concerning hematological malignancies reported in hematology journals with a high impact factor. The secondary goals were to address whether outcome reporting discrepancies favored statistically significant outcomes, whether a pattern existed between the funding source and likelihood of outcome reporting bias, and whether temporal trends were present in outcome reporting bias. We also catalogued incidental findings during data extraction and analysis that warranted further examination. Following extraction and analysis of data, we emailed the authors of RCTs with major discrepancies to determine the reason for outcome changes from registration to publication. This study did not meet the regulatory definition of human subjects research according to 45 CFR 46.102(d) and (f) of the Department of Health and Human Services’ Code of Federal Regulations [17], and it was not subject to Institutional Review Board oversight.

We consulted Li et al [18] the Cochrane Handbook for Systematic Reviews of Interventions [19]; and the National Academies of Science, Engineering, and Medicine’s Standards for Systematic Reviews [20] to ensure best practices regarding data extraction and management. We applied relevant PRISMA guidelines [21] to ensure reporting quality for systematic reviews and SAMPL guidelines [22] for reporting descriptive statistics. This study was registered with the University hospital Medical Information Network Clinical Trials Registry (UMIN-CTR) prior to commencement (R000025787UMIN000022374). Data for this study are available on figshare (https://doi.org/10.6084/m9.figshare.4968476). A PRISMA Checklist is available for this study as a supplemental file (S1 Checklist).

### Eligibility criteria for considering studies for this review

We used the Google H-5 power index to identify relevant hematology journals. Journals were chosen based off whether or not they publish RCTs concerning hematological malignancies, according to their “About” section on their respective websites. RCTs published in *Blood*; *British Journal of Haematology*; *American Journal of Hematology*; *Leukemia*; and *Haematologica* were included. We searched for RCTs indexed in PubMed between January 1, 2010, and December 31, 2015. This time period was several years after the ICMJE trial registration policy and allowed sufficient time to observe reporting trends. We used the National Institutes of Health definition of clinical trial: “a research study in which one or more human subjects are prospectively assigned to one or more interventions (which may include placebo or other control) to evaluate the effects of those interventions on health-related biomedical or behavioral outcomes [23].” We included RCTs, RCTs that used a crossover method, and follow-up studies if the listed trial registration number was for the follow up only, rather than for the primary analysis also. This ensured that the outcomes were properly evaluated for changes.

### Search strategy for identifying relevant studies

Our search was performed with assistance from a medical research librarian with the following search string: (("Br J Haematol"[Journal] OR "Haematologica"[Journal]) OR "Leukemia"[Journal] OR "Blood"[Journal]) OR "Am J Hematol"[Journal] AND (Randomized Controlled Trial[ptyp] AND ("2010/01/01"[PDAT]: "2015/12/31"[PDAT])). The search occurred on September 26, 2016.

### Study selection and data extraction

Citations retrieved during the search were uploaded to Endnote X7.5. Two investigators independently screened the title and abstract of each citation for possible inclusion after completing an internally developed training exercise. Any disagreement about inclusion was resolved by consensus. Excluded citations were copied into Excel and coded for the reason for exclusion. Investigators were blinded to a trial’s registration status during screening to minimize observer bias.

After screening, citations were imported into the Agency for Healthcare Research and Quality’s Systematic Review Data Repository (SRDR) [24] for data extraction. For calibration and for minimizing discrepancies in extraction, each investigator underwent SRDR and data extraction training following an internally developed protocol.

Two investigators independently reviewed the full-text articles. Once per day, these investigators met to resolve disagreements. A third investigator was available for adjudication but was not needed. We extracted the following items from the published RCT: primary outcome(s), secondary outcome(s), subject enrollment date, trial registry database and registration number, timing of assessment in primary outcomes, sample size, any discrepancies between publication and registry disclosed by the author, and funding source. We classified funding source into the following categories: private, public, industry/corporate, mixed funding, or undisclosed. For RCTs that reported multiple primary and secondary outcomes, we recorded each explicitly stated outcome. If authors failed to differentiate between primary and secondary outcomes in the publication, these non-delineated outcomes were coded as “unspecified,” set aside for individual analysis, and excluded from selective reporting analysis.

If a publication did not discuss registration, we emailed authors and asked about registration status. If we did not receive a reply after 1 week, a second email was sent. If trialists did not respond to email attempts, we designed search queries for each trial with unknown registration status. For ClinicalTrials.gov queries, we used every author's last name listed on the publication, separated them using the Boolean operator "OR", and placed the string in parentheses. We next selected key words from each title (such as the intervention and condition) that were more likely to generate accurate search returns and used the same Boolean operator and parenthetical organization. The author and keyword stings were next joined by the Boolean operator "AND". For the WHO ICTRP, each search was modified to accommodate the particular search capabilities of this registry. We consulted Glanville et al. [25] to appropriately translate our search queries. After both sets of search queries were built, the search was performed.

Trial data were extracted from registries by two independent investigators. The following data were extracted using SRDR: date of trial registration, date range of subject enrollment, original primary registered outcome(s), final primary registered outcome(s), date of initial primary outcome registration, secondary registered outcome(s), sample size, and funding source using previously defined categories. Trials lacking registration of primary or secondary outcomes and those registered after the completion of the trial (retrospectively) were excluded from our primary analysis. Per the International Standards for Clinical Trial Registries section 2.4, [26] WHO-approved registries are required to time-stamp registry-approved changes to any registered trial, including data additions, deletions, and revisions. Therefore, if a WHO-approved trial registry did not display a history of changes, we recorded the date the registry application was approved as the date of initial primary outcome registration. We used the same methodology for other non-WHO registries with the same conditions.

Three investigators next compared primary and secondary outcome(s) listed in the publication to the initial registered primary outcome(s) for consistency. Decisions were made by consensus. We catalogued 5 major discrepancies according to the classification system described by Chan et al [27] and refined by Mathieu et al [15]:

1. A registered primary outcome was demoted to secondary in the publication.
2. A registered primary outcome was omitted from the publication.
3. A new primary outcome was silently added to the publication.
4. A registered secondary outcome was promoted to primary in the publication.
5. The timing of assessment of the registered and published primary outcomes differed.

We also noted other discrepancies. These included instances of a demoted or omitted secondary outcome and a silently added unspecified outcome.

Articles with discrepancies were also assessed to determine whether the discrepancies favored statistically significant results. Examples of a statistically significant discrepancy include a silently added primary outcome or a promoted secondary outcome, each with a *P*-value less than 0.05. Additionally, if a primary outcome was omitted or demoted with a *P*-value greater than 0.05, this was also considered significant.

Data were exported from SRDR and analyzed using Excel 2013. We used STATA 13.1 (StataCorp, College Station, Texas) for statistical analysis. We performed a Fisher’s exact test to evaluate the relationship between funding source and selective outcome reporting for trials that reported *P*-values.

Following data analysis, we emailed the corresponding author of all trials for which a major discrepancy was found. In the email, we first listed all changes between the registry-listed outcomes and the published outcomes. We asked authors to verify that our data were accurate. We next asked authors to disclose the reasons for outcome changes. The response options were based on previous literature [28–30]. This email also contained a link to a Google Form which also listed the potential reasons for outcome changes in case authors preferred to answer in this manner. We employed Dillman’s method of contacting trialists three times over 1 week intervals to improve response rates [31]. The email template is available on figshare. (https://doi.org/10.6084/m9.figshare.4968476).

## Results

**Fig 1:**
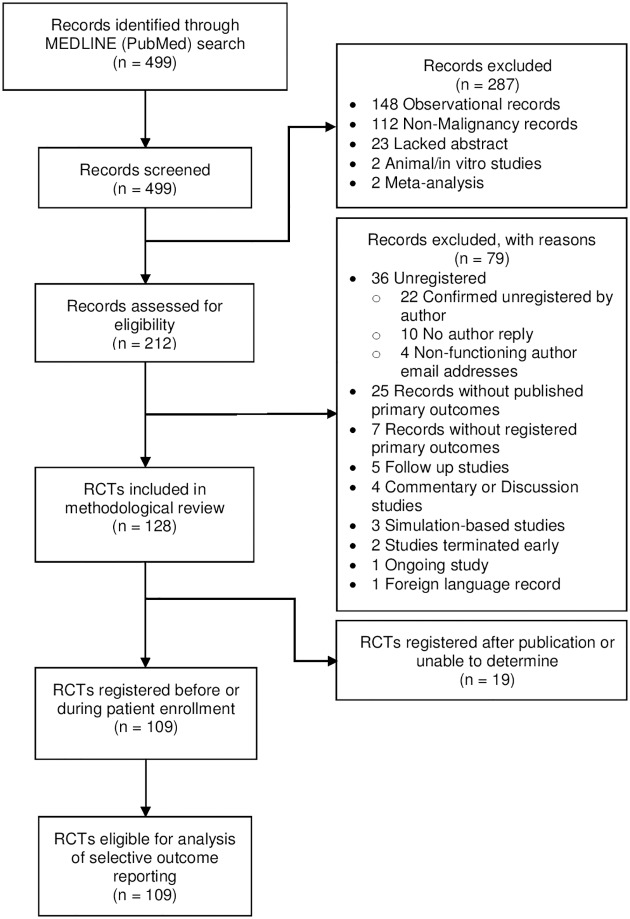
PRISMA flow diagram. Details regarding exclusions at each level of analysis.

Our search yielded 499 records. Excluded records and the reason for exclusion are shown in the PRISMA flow diagram (Fig 1). One hundred twenty-eight RCTs were eligible for inclusion. One hundred nine (85.2%) RCTs were registered before completion of the trial and constituted our final sample.

The demographics of included RCTs are listed in Table 1. We encountered 6 different registries in our analysis. The majority of trials were registered in ClinicalTrials.gov followed by ISRCTN (Table 1).

**Table 1:** Demographic information. Characteristics of included RCTs published between January 1, 2010 and December 31, 2015.

n = 109	
**Journal**	
*Blood*	51
*American Journal of Hematology*	5
*British Journal of Haematology*	23
*Leukemia*	6
*Haematologica*	24
**Funding Source**	
Industry/Corporate	45
Private	8
Public	20
Mixed	33
None Disclosed	3
**Registry**	
Australian New Zealand Clinical Trials Registry	1
ClinicalTrials.gov	90
EU Clinical Trials Register	3
ISRCTN	10
Netherlands Trial Registry	3
UMIN	2
**Time of Registration**	
Prospective	83
During Patient Enrollment	26
**Publication Year**	
2010	15
2011	11
2012	18
2013	19
2014	20
2015	26
**Total RCTs Stratified by Number of Major Discrepancies**	
0	56
1	20
2	21
3	3
4+	9

The primary outcome was the number of major discrepancies present in our sample. The 109 RCTs evaluated included 118 major discrepancies, and 629 discrepancies overall. Among the 118 major discrepancies, 30 (25.4%) primary outcomes were demoted, 47 (39.8%) primary outcomes were omitted, and 30 (25.4%) primary outcomes were added. Three (2.5%) secondary outcomes were upgraded to a primary outcome. The timing of assessment for a primary outcome changed eight (6.8%) times. Fifty-six RCTs (51.4%) were reported without major discrepancy and 11 (10.1%) were reported without discrepancy whatsoever. Two (3.6%) of the RCTs without a major discrepancy made changes to their registered outcomes after publication. The frequency of major discrepancies over time shows no detectable trend (Table 2).

**Table 2:** Number of major discrepancies per year. Frequency of major discrepancies between registered and published outcomes of included RCTs (n = 109) and whether discrepancies favored statistical significance, by year.

Publication Year	Number of major discrepancies	Number of discrepancies with a reported P-value		Of evaluable discrepancies, number of discrepancies favoring statistically significant results	
2010	18	3	17%	3	100%
2011	19	4	21%	3	75%
2012	13	4	31%	3	75%
2013	27	5	19%	5	100%
2014	16	5	31%	3	60%
2015	25	10	40%	8	80%
Total	118	31	26%	25	81%

Thirty-one major discrepancies were published with a *P*-value; twenty-five (80.6%) favored statistical significance. Results from Fisher’s exact test support a relationship between funding source and selective outcome reporting among trials with reported *P*-values (p = .023). Detailed information regarding discrepancies by funding source is shown in Table 3.

**Table 3:** Number of major discrepancies per funding source. Published RCTs that were registered before or during trial completion and have major discrepancies with their trial registries and the effect of these discrepancies on the statistical significance of published outcomes, by funding source.

Number of published RCTs	Total		*Private*		*Public*		*Industry*		*Mixed*		*Undisclosed*	
	109		8		20		45		33		3	
Number of published RCTs with major discrepancies between registry and publication	53	49%	2	25%	12	60%	17	38%	19	58%	3	100%
Number of major discrepancies between registry and publication	118		3		32		36		40		7	
Registered primary outcomes demoted in publication	30	25%	0	0%	12	38%	6	29%	12	30%	0	0%
Registered primary outcomes omitted from publication	47	40%	1	33%	12	38%	14	39%	14	35%	6	86%
Unregistered primary outcomes added to publication	30	25%	1	33%	6	19%	13	36%	9	23%	1	14%
Registered secondary outcomes promoted in publication	3	3%	0	0%	1	3%	2	6%	0	0%	0	0%
Timing of assessment of primary outcomes differs	8	7%	1	33%	1	3%	1	3%	5	13%	0	0%
Number of major discrepancies between registry and publication	118		3		32		36		40		7	
Did not report p-values	87	74%	1	33%	21	66%	27	75%	32	80%	6	86%
Reported p-values	31	26%	2	67%	11	34%	9	25%	8	20%	1	14%
Major discrepancy favors statistical significance	25	81%	2	100%	11	100%	5	56%	7	88%	0	0%
Major discrepancy does not favor statistical significance	6	19%	0	0%	0	0%	4	44%	1	13%	1	100%
Number of RCTs containing major discrepancies favoring statistical significance	19		2		6		5		6		0	

Our final analysis included an email to the authors of the 53 RCTs with a major outcome discrepancy between registry and publication. One RCT [32] already mentioned a protocol change in their publication; therefore, 52 authors were emailed. The response rate was 23.1% (12/52). Two authors had non-functioning email addresses. The most common reason for a change in outcomes was a pre-planned extension study or subgroup analysis (5/12, 41.7%). Two authors (16.7%) denied a change in outcomes, despite our findings, but did not elaborate further. One author admitted to a change occurring after discussion with peer reviewers. One author reported a change in outcome following the publication of new clinical evidence which resulted in a change to best practices, and one author cited insufficient power to analyze the outcome. One author replied that our study revealed a mistake in trial registration that resulted in a different timing of assessment in the registry compared to the original protocol. This error was corrected in the registry on April 18, 2017.

## Discussion

Our results suggest that selective outcome reporting bias may occur frequently in hematology journals. Across trials, we found 118 major discrepancies and 629 discrepancies overall. Furthermore, 80.6% of major discrepancies with a reported *P*-value (n = 31) favored statistically significant results. In addition to major discrepancies, authors often contributed to other discrepancies, seemingly treating non-primary outcomes as malleable. These results indicate a need within hematology for heightened attention to timely and consistent registration of outcomes by both authors and journals alike, as has been indicated in other medical specialties [15, 33]. Because RCTs ultimately underpin clinical judgment and guide policy implementation, selective reporting of outcomes threatens medical decision making.

Other studies of selective outcome reporting across various fields of medicine support our findings [15, 27, 34–37]. The vast majority (85.2%) of hematology RCTs we evaluated were registered before the end of patient enrollment. Hematology RCTs were properly registered more so than has been reported in other specialties [15, 36, 37]. However, the rates of selective reporting bias found in this investigation are cause for concern and appears to be a significant issue in many medical specialties.

Adequate trial registration and adherence to reporting guidelines can potentially limit selective outcome reporting; however, it must be the joint effort of authors, editors, peer reviewers, and other stakeholders. Authors must adhere to guidelines and use best practices to improve the quality of their studies, which includes prospectively registering trials, clearly defining primary and secondary outcomes, detailing ethical changes to the registry during their study period, and addressing discrepancies in their published reports. Editors and peer reviewers should use registries to evaluate manuscripts for accuracy of data and consistency of outcomes and to verify adequate registration prior to publication. Another option would be to design registry databases so that trial registration could not be completed without first listing pertinent information like methodology, primary and secondary outcome(s), and date of participant enrollment. Some methods to improve outcome reporting have already been instituted, such as a declaration of transparency by the lead author, which has been adopted by *The BMJ* and *BMJ Open*, stating that the manuscript shows an honest and accurate account of the study. Finally, funding agencies should take responsibility by auditing the consistency and completeness of trial results and making authors accountable for discrepancies with regard to funding for subsequent research [38].

Prior studies have not identified a correlation between frequency of discrepancies and funding source [5, 13, 27]. In our sample, publicly funded RCTs had a higher frequency of major discrepancies that favored statistical significance; however, the reasons for this finding are not clear. Action is being taken to improve aspects of trial registration and reporting. Effective January 18, 2017, the National Institutes of Health (NIH) began requiring all RCTs funded, in any part, by the NIH to be registered in ClinicalTrials.gov and to report summary results and adverse events [39]. This new requirement will likely improve rates of reporting and potentially reduce rates of selective reporting bias.

Trial registration aims to enhance transparency and accountability in planning, conducting, and reporting clinical trials by making details about a trial available to the public [9]. In 2005, ICMJE instituted a policy requiring prior registration as a condition for publication. The Food and Drug Administration later mandated that all applicable clinical trials be prospectively registered. Our data reflect this policy shift; however, we still found evidence of retrospective registration. Overall, the rate of trial registration has improved, with a 5-fold increase in global trial registration from 2004 to 2013 [40]. According to their Instructions for Authors sections, *Blood*, *Leukemia*, *and Haematologica* all require registration of clinical trials prior to patient enrollment, adherence to the ICMJE’s Uniform Requirements for Manuscripts (URM), and CONSORT guidelines. *British Journal of Haematology* and *American Journal of Hematology* require adherence to the ICMJE’s URM but do not mention adherence to CONSORT guidelines or trial registration. Item 6a on the CONSORT checklist and the ICMJE’s URM section III.L requires authors to completely define pre-specified outcomes in their publication.

The most frequent major discrepancy encountered was the omission of a registered primary outcome from the publication. We found that, on average, there were more than four publications associated with each trial registration number in our sample. These two findings must be interpreted together. We understand that many trialists report preliminary data or subgroup analyses which are dispersed over multiple publications, and this is supported by the results of our survey. This multiplicity might account for some of the omitted primary outcomes in our sample. We were unable to determine which specific outcomes were omitted completely and which were described in other publications. We, therefore, recommend that hematology trialists fully describe the extent to which outcomes are reported elsewhere. Duplicate publication and “salami slicing” [41–48] are noted concerns in medicine and would be an interesting line of future research in hematology.

The difficulty in determining which omitted outcomes were published elsewhere and which were truly omitted emphasizes a potential shortcoming in trial registries. This issue is also a limitation of our study. These registry shortcomings include, but are not limited to, permitting retrospective registration and not providing dedicated fields in the registries for authors to detail pre-planned subgroup analyses. A recent Special Report in the *New England Journal of Medicine* highlights these concerns [49]. The authors of this report correctly state that allowing retrospective trial registration fundamentally undermines the primary goal of public trial registrations by interfering with the ability to see the evolution of trial outcomes and the amount of unpublished data that were generated. They further note the difficulty in determining which major discrepancies are benign (e.g., an outcome was omitted from publication because it was published in the subgroup report) with respect to pre-planned subgroup analyses. We encountered this problem when we emailed authors. We found five confirmed discrepancies due to pre-planned subgroup analyses. In our study, outcome discrepancies due to subgroup analyses should be considered when interpreting our results. Concerns regarding subgroup analyses are widespread [50–54] and revolve around statistical methods, statistical power, and credibility of subgroup analysis claims. Mandatory reporting of subgroup analyses outcomes and methodology would allow for the proper evaluation of selective reporting bias and improve transparency. The standardization of study protocols [55] and the ability to clarify pre-planned subgroup outcomes in trial registries are reasonable steps toward reducing selective reporting bias.

We acknowledge that during the course of a clinical trial, adaptations may need to be made due to unforeseen toxicities or changes in study design. For example, one RCT measured progression-free survival in patients with CD-20-positive diffuse large B-cell lymphoma randomized to receive rituximab, cyclophosphamide, doxorubicin, vincristine, and prednisone plus either placebo (R-CHOP) or bevacizumab (RA-CHOP). Early analysis revealed poor risk/benefit ratios of RA-CHOP compared to R-CHOP alone, with elevated rates of cardiotoxicity in the group receiving RA-CHOP. The trial was terminated early, and safety with 12 month follow up became the primary end point. This RCT provides an example of the need to alter the trial due to unforeseen circumstances, and in such cases, changes to the registry record are needed [56]. For trials that continue despite changes in protocol, proper disclosure of protocol changes should coincide with an immediate update of the trial registry. This includes updating the registry to reflect changes to outcomes, including the timing of assessment.

To conclude, selective outcome reporting continues to be prevalent. In many cases, authors are inhibited by shortcomings in trial registries and are unable to clarify how their outcomes are allocated (e.g., as primary or subgroup analysis). Joint efforts to ensure publication quality and unbiased results require authors, editors, and reviewers to all participate in the process. Such collaboration will bolster the accuracy and reliability of outcomes and therefore that of trials, clinical decision making, and health policy.

## Supporting information

S1 Checklist PRISMA item checklist. List of relevant PRISMA Items with corresponding page number denotation.
(DOCX)
